# Antimicrobial susceptibility profile of selected Enterobacteriaceae in wastewater samples from health facilities, abattoir, downstream rivers and a WWTP in Addis Ababa, Ethiopia

**DOI:** 10.1186/s13756-019-0588-1

**Published:** 2019-08-09

**Authors:** Hemen Tesfaye, Haile Alemayehu, Adey F. Desta, Tadesse Eguale

**Affiliations:** 1 Department of Food Process Engineering, Dire Dawa University, P.O. Box 1362, Dire Dawa, Ethiopia; 2 Aklilu Lemma Institute of Pathobiology, Addis Ababa University, P.O. Box 1176, Addis Ababa, Ethiopia; 3 Department of Microbial, Cellular and Molecular Biology, College of Natural and Computational Sciences, Addis Ababa University, P.O. Box 1176, Addis Ababa, Ethiopia

**Keywords:** Antimicrobial resistance, Enterobacteriaceae, Wastewater, Hospital, Abattoir, River

## Abstract

**Background:**

Evaluation of antimicrobial susceptibility profile of various bacterial pathogens in the health facilities, abattoirs and related environment is important to assess potential risk of dissemination of resistant pathogens to the environment. There is limited information about antimicrobial susceptibility profile of common Enterobacteriaceae in waste water samples from hospitals, abattoirs and the downstream water bodies in Addis Ababa. The present study assessed antimicrobial susceptibility of bacteria belonging to the family Enterobacteriaceae isolated from wastewater samples (WWS) of two hospitals: Tikur Anbessa Specialized Hospital (TASH) and Minilik II hospital, a wastewater treatment plant (WWTP) and an abattoir, and downstream rivers in Addis Ababa.

**Results:**

A total of 54 bacterial isolates belonging to 6 species were identified: *E.coli* (32%), *Salmonella* 23%), *Klebsiella pneumonia* (15%), *Enterobacter aerogenes* (11%), *Citrobacter* (7%), *Klebsiella oxytoca* (6%) and *Enterobacter cloacae* (6%), respectively. Two strains of Citrobacter spp. isolated from TASH wastewater sample (WWS) were resistant to all 12 antimicrobials tested whereas an *E. coli* isolate from the same source was resistant to 11 antimicrobials. All isolates were resistant to 2 or more antimicrobials tested. Multi-drug resistance (MDR) to several antimicrobials was recorded, particularly in isolates obtained from hospital WWS and it was more common in *Citrobacter* and *E. coli* isolates. Extended spectrum betalactamase (ESBL) production was detected in 27.3% of MDR isolates, all of them obtained from hospital effluents whereas none of the isolates were carbapenemase producers.

**Conclusion:**

The present study revealed that Enterobacteriaceae in wastewater from hospitals, abattoir and downstream water bodies are resistant to commonly used antimicrobials. Hospital effluents contained more of MDR bacteria, posing significant public health threat through dissemination to the downstream water bodies.

## Background

Antimicrobials are natural or synthetic chemicals that kill or inhibit the growth of susceptible microorganisms. They have been used to treat and prevent microbial infections in human and veterinary medicine for decades [1]. Misuse and overuse of antimicrobials in humans and livestock has led to the emergence of antimicrobial resistant bacterial strains compromising the effectiveness of antimicrobial therapy [2, 3]. Hospitals are the primary hotspots for selection of resistant microbes where several types of antimicrobials are flushed frequently inducing high selection pressure in the bacterial community [4]. In addition, the misuse and overuse of antimicrobial agents in food animal production are also posing serious risk of selection for resistant bacterial pathogens and commensal organisms [5]. In developing countries like Ethiopia, wastewater generated from most of the hospitals and abattoirs do not get appropriate treatment before being released to the nearby rivers and streams. The effluent could contain multi-drug resistant (MDR) pathogenic organisms capable of causing infection in humans and animals or commensal organisms capable of transmitting their resistance genetic markers to other bacterial species in the environment impacting natural ecosystem [6]. The discharge of untreated wastewater to the water bodies greatly contributes to the environmental pool of antimicrobial resistant bacteria and antimicrobial resistance genes [7–9].

In Addis Ababa, wastewater from some of the health facilities including hospitals, livestock farms and abattoir facilities are directly discharged to the rivers. The contaminated rivers are usually used for irrigation purpose by small scale farmers growing vegetables. This may serve as source of infection with resistant pathogenic bacteria to the farmers themselves and consumers of the vegetables. Abattoirs in most developing countries have unhygienic environments that promote the growth of pathogenic microorganisms [10]. In Addis Ababa, some of the animals are sourced from feedlots where antimicrobials are used frequently creating selection pressure on susceptible bacterial communities. In addition, some of the livestock traders treat animals with broad spectrum antimicrobials before transportating to abattoir to protect animals from stress associated infections, leading to increased risk of release of antimicrobial resistant bacteria in the abattoir environment and wastewater from such facilities. Recent study in Ethiopia showed high residue level of tetracycline in slaughtered beef cattle from three slaughter houses in central Ethiopia [11]. Untreated effluent of Addis Ababa abattoir flows in to Little Akaki river directly without any wastewater treatment.

In Ethiopia, particularly, in Addis Ababa, there is no data concerning resistance profile of microorganisms isolated from hospital wastewater, abattoirs and downstream water bodies. Assessing antimicrobial susceptibility of bacterial isolates from such environmental samples will give clue on the potential risk of dissemination of antimicrobial resistance to the downstream ecology and its public health consequences. This study is therefore designed to determine occurrence and antimicrobial susceptibility profile of common pathogenic bacterial species belonging to family Enterobacteriaceae in wastewater originating from hospitals and an abattoir as well as downstream water bodies used to irrigate small scale farms in Addis Ababa, Ethiopia.

## Methods

### Study area

The study was conducted in Addis Ababa, capital city of Ethiopia. Wastewater samples released from Menilik II Hospital, Tikur Anbessa Specialized Hospital (TASH), and Addis Ababa Abattoir were the primary sampling sources. In addition, downstream water bodies: Kebena river contaminated with wastewater from Minilik II Referral Hospital, and Little Akaki river contaminatedwith wastewater from Addis Ababa Abattoir were also sampled. These rivers were used by small scale farmers for irrigation to grow vegetables. Menilik II Referral Hospital has 29 active specialized case teams with different outpatient and inpatient Departments. Wastewater generated from the hospital was directly discharged to Kebena River when this study was conducted. Tikur Anbessa Specialized Hospital (TASH) is located at the center of Addis Ababa and it is the largest referral hospital in the city and is the main teaching hospital for both clinical and preclinical training of College of Health Sciences, Addis Ababa University and also provides referral services to the community in Addis Ababa as well as other regions of the country. The wastewater sources generated from the hospital including students’ dormitory, toilets, laboratories and cafeterias flow directly to the main sewage line that joins Kality wastewater treatment plant. Based on personal communication with the hospital communities, the hospital does not have functional onsite treatment plants at the time of sampling.

**Fig. 1:**
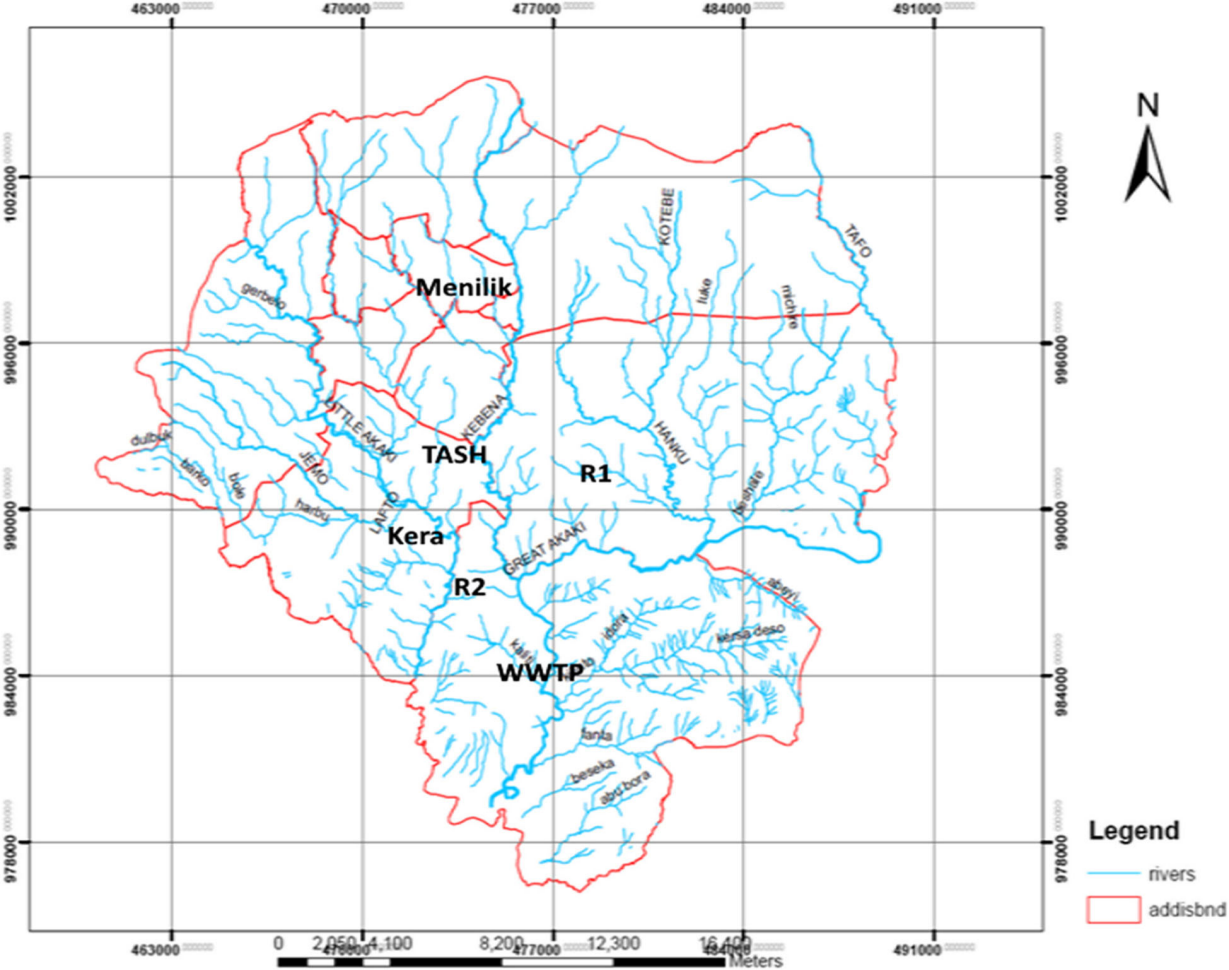
Sampling points from the hospitals, abattoir, rivers and a wastewater treatment plant in Addis Ababa (TASH:Tikur Anbessa Specialized Hospital; R1: Kebena River; Kera: Addis Ababa Abattoir; R2: Little Akaaki river; WWTP: Kality wastewater treatment plant). (Source: Image obtained from Addis Ababa Planning Commission, 2016

The Addis Ababa Abattoir located in central Addis Ababa is the largest abattoir in the city providing majority of the city’s meat demand. The abattoir on average gives slaughter service for about 400 goats, 600 sheep and 700 cattle daily. The abattoir uses large volume of water for washing meat and cleaning processing areas. This large consumption of water leads to generation of a significant volume of wastewater, which is directly discharged in to Little Akaki River without any prior treatment [12]. Kaliti WWTP is located in the southern part of Addis Ababa. The wastewater from housing units and TASH connected to the city’s sewer system is conveyed to this treatment plant. Figure 1 shows map of Addis Ababa and specific sample collection sites.

### Sample collection and isolation of selected bacteria

A total of 24 wastewater samples were collected from the 6 collection points in two rounds in November and December 2017. Duplicate wastewater samples from each site were collected using 200 ml sterile bottles. The samples were then transported to Microbiology Laboratory, Aklilu Lemma Institute of Pathobiology, Addis Ababa University within 3–4 h of collection in an ice box. Isolation of members of the family Enterobacteriaceae was conducted according to standard procedures using general, selective and differential media. For isolation of Klebsiella Spp., *Escherichia coli*, Enterobacter Spp. and Citrobacter Spp*.* loopful of the sample suspension was inoculated using sterile inoculating loop on pre-dried sterile Eosin methylene blue (EMB) agar plates. Each sample was inoculated on two petridishes and a single colony with distinct feature representing a given species of Enterobacteriaceae was picked and stored for further characterization from each plate. The plates were incubated at 37 °C for 24 h. For isolation of *Salmonella,* 10 ml of wastewater sample was inoculated in to 90 ml of buffered peptone water (BPW) and incubated for 24 h at 37 °C. Then 100 μl of this suspension was transferred to 10 ml of Rappaport-Vassiliadis enrichment broth (RVB) and incubated for 24 h at 37 °C and 1 ml of suspension was transferred to 10 ml of Tetrathionate broth (TTB) and incubated for 24 h at 42 °C. The samples from these two broths were streaked on to Xylose Lysine tergitol 4 (XLT-4) selective media and the plates were incubated at 37 °C for 24 h [13]. Presumptive *Salmonella* colonies were then inoculated in to tryptone soya agar (TSA) slant and grown over night at 37 °C and kept for further analysis.

Distinct presumptive colonies of each suspected bacterial species were picked and confirmed using various biochemical tests on Urea agar, Lysine iron agar, Citrate agar, Triple sugar iron agar slants and motility test was also conducted. In addition, Citrobacter, Klebsiella, and Eterobacter spp.were confirmed using API 20E kit (Analytical Profile Index), a biochemical panel for identification and differentiation of members of the family Enterobacteriaceae and the software APIWEB (Biomérieux, France) was used to interpret the result of the reading after incubation of the organism in each chamber according to the instruction provided by the manufacturer. Isolates were tested for Gram positivity and Gram negativity using 3% KOH [14]. *Salmonella* isolates were further confirmed by genus specific PCR as described previously [15]. In addition, PCR confirmed *Salmonella* isolates were serotyped at the National Microbiology Laboratory, *Salmonella* Reference Laboratory, Public Health Agency of Canada as described previously based on types of somatic (O) and flagellar (H) antigens of the *Salmonella* isolates using slide agglutination test [16, 17].

### Antimicrobial susceptibility of bacterial isolates

Antimicrobial susceptibility of bacterial isolates to a panel of 12 antimicrobials was determined using disc diffusion assay according to the Clinical and Laboratory Standards Institute guidelines [18] on Mueller Hinton Agar plate (Oxoid, Ltd). The following antimicrobials (Sensi-Discs, Becton, Dickinson and Company, Loveton, USA) and disc potencies were used: amoxicillin + clavulanic acid (Amc) (20/10 μg), ampicillin (Amp) (10 μg), cefoxitin (Fox) (30 μg), ceftriaxone (Cro) (30 μg), cephalothin (Cf) (30 μg), ciprofloxacin (Cip) (5 μg), gentamicin (Gm) (10 μg), streptomycin(S) (10 μg), sulfisoxazole (G) (1000 μg), sulfamethoxazole + trimethoprim (Sxt) (23.75/1.25 μg), trimethoprim (Trm)(5 μg) and tetracycline (Te) (30 μg). The interpretation of the categories of susceptible, intermediate or resistant was based on the CLSI guidelines [18]. Isolates were regarded as multi-drug resistant (MDR) when they were resistant to at least three or more antimicrobial classes. *E. coli* ATCC 25922 was used as a quality control organism.

### Investigation for extended spectrum betalactamase production

Double-disc synergy test (DDST) was used to detect production of extended spectrum betalactamase among selected Enterobacteriaceae (*n* = 22) which exhibited intermediate susceptibility or full resistance to second and/or third generation cephalosporins (cefoxitin and ceftriaxone) as described previously [19]. The test isolates were grown in Muller Hinton Broth for 3–4 h and standardized to equal turbidity level of 0.5 Mac Ferland standards. It was swabbed on a surface of Mueller-Hinton agar plate on which 30 μg ceftriaxone disc and amoxicillin+clavulanic acid disc (20 μg/10 μg) was placed at a distance of 20 mm center to center. The plates were incubated at 37 °C overnight and zone of inhibition was measured. The bacterial isolates were considered positive for the ESBL production when a decreased susceptibility to ceftriaxone was combined with a clear-cut enhancement of the inhibition zone of ceftriaxone in front of the clavulanate-containing disc [20].

### Modified carbapenem inactivation assay

Carbapenemases are broad spectrum betalactamases that hydrolyze carbapenems as well as other betalactams. Modified carbapenem inactivation assay was used to assess production of carbapenemase in suspected Enterobacteriaceae as described previously using 10 μg meropenem disc and known meropenem susceptible reference strain of *Escherichia coli* ATCC 25922 [21].

### Data analysis

Data was entered to Microsoft Excel and descriptive statistics such as percentage were used to analyze the data.

## Results

### Distribution of bacterial genera isolated from the wastewater samples

A total of 54 isolates belong to five genera and 7 species of the family Enterobacteriaceae were isolated from the six wastewater sites during the two sampling times. Among all bacterial species, *E. coli* was dominantly isolated from 75% of samples, comprising 31.5% of the total isolates. The second most commonly isolated member of the family Enterobacteriaceae was Salmonella Spp. which was isolated from WWSs from TASH, Kality WWTP, Addis Ababa Abattoir and Little Akaki river comprising 24.1% of the total isolates. *Klebsiella pneumoniae* was the third most dominant species comprising 14.8% and it was isolated from WWSs from Menilik II Referral Hospital, Addis Ababa Abattoir and Little Akaki river. *Enterobacter cloacae* was the fourth dominant species comprising 11.1% of the total isolates. It was isolated from two of the six sites namely Addis Ababa abattoir and Little Akaki river (Table 1). Although *Salmonella* was isolated from all study sites, recovery of *Salmonella* was more common in samples from downstream rivers and Addis Ababa abattoir than the two hospital WWSs.

**Table 1:** Overall rate of isolation of members of the family Enterobacteriaceae from hospitals and abattoir wastewater, their receiving rivers and Kality WWTP

No.	Species	No. isolated (%)	Place of collection and number isolated
1	*E. coli*	18 (75)	TASH(4), Minilik II HospitalWWS(3), AA abbatoir(4), Kality WWTP(2), Kebena River(1),Little Akaki river(3)
2	Salmonella spp.	12 (50)	Minilik II Hospital WWS(1), TASH WWS (1), A. A Abattoir WWS(2), Little Akakki river (3) Kality WWTP(2), Kebena river(3)
3	*Klebsiella pneumoniae*	8 (33.3)	Minilik II Hospital WWS(2), A. A Abattoir WWS(1), Kality WWTP(2),
4	*Klebsiella oxytoca*	3 (12.5)	TASH WWS(1), Minilik II Hospital WWS(1), Little Akaki river(1)
7	*Enterobacter cloacae*	6 (25)	TASH WWS(2), Minilik II Hospital WWS(1), kebena river(1), Kality, WWTP(1), Little Akaki river(1)
5	*Enterobacter aerogenes*	3 (12.5)	Little Akakiriver(1), AA abattoir(2)
6	Citobacter Spp.	4 (16.7)	TASH WWS(2), Kality WWTP(1), Little Akaki river(1)

### Serotype distribution of *Salmonella* isolates

Among the 12 *Salmonella* isolates recovered in the current study, *S*. Muenchen(*n* = 4), *S*. Typhimurium(*n* = 3), *S*. Newport(*n* = 2) were the dominant serotypes. *S*. Adelide, *S*. Cerro and *S.* Parathyphi B (*n* = 1) were also recovered (Table 2). To our knowledge, *S.* Adelide has not been reported from Ethiopia previously.

**Table 2:** *Salmonella* serotype distribution

Isolate ID	Serotype	Antigenic formula	Place of collection
H7	*S.* Newport	6,8:e,h:1,2	AA Abattoir WWS
H10	*S.* Newport	6,8:e,h:1,2	TASH WWs
H13	*S.* Typhimurium	4,5:i:1,2	Minelik II Hospital WWs
H18	*S.* Parathyphi B	4:b:1,2	AA Abattoir WWS
H36	*S*. Muenchen	6,8:d:1,2	Little Akaki river
H41	*S*. Cerro	6,14,18:z4,z23: [1, 5]	Kaity WWTP
H46	*S*. Typhimurium	4,5:i:1,2	Kebena river
H47	*S.* Muenchen	6,8:d:1,2	Kebena river
H48	*S.* Muenchen	6,8:d:1,2	Little Akaki river
H58	*S*. Adelide	35:f,g:-	Little Akaki river
H59	*S*. Muenchen	6,8:d:1,2	Kaity WWTP
H61	*S.*Typhimurium	4,5:i:1,2	Kebena river

### Antimicrobial susceptibility profile of bacterial isolates

Susceptibility of bacterial isolates to different antimicrobials is shown Table 3. Bacterial isolates collected from all the 6 sampling sites were totally resistant to ampicllin and cephalothin. Of all *E. coli* isolates 15(83.3%) were resistant to amoxicillin/clavulanic acid, and all isolates from TASH WWS were resistant. Resistance to cefoxitin (2^nd^ generation cephalosporin) was detected in 10 (55.6%) of the *E. coli* isolates whereas resistance to ceftriaxone (3^rd^ generation cephalosporin) was detected in 5(27.8%) of the isolates in which three of the resistant isolates were from the two hospital WWSs and the two other isolates were from Little Akaki river. The overall rate of resistance to majority of antimicrobials was high in *E. coli* isolates obtained from the two hospital WWSs, followed by isolates from Addis Ababa Abattoir WWS.

**Table 3:** Bacterial species isolated and rate of occurrence of resistance to selected antimicrobials

Species of bacteria	*No. tested*	Number and (%) resistant to antimicrobials tested											
		Amc	Cip	Gm	S	Sxt	Trm	Te	Amp	Cf	Fox	Cro	Su
*Escherichia coli*	18	15 (83.3)	5 (27.8)	–	–	–	7 (38.9)	13 (72.2)	18 (100)	18 (100)	10 (55.6)	5 (27.8)	6 (33.3)
Salmonella spp.	12	3 (25)	–	–	3 (25)	1 (8.3)	1 (8.3)	7 (58.3)	12 (100)	12 (100)	7 (58.3)	0	2 (16.7)
*klebsiella pneumoniae*	8	5 (62.5)	1 (12.5)	–	–	2 (12.5)	2 (25)	3 (37.5)	ND	ND	4 (50)	2 (25)	3 (37.5)
*Klebsiella oxytoca*	3	3 (100)	–	–	–	–	–	–	ND	ND	–	–	–
*Enterobacter cloacae*	6	5 (83.3)	3 (50)	1 (16.7)	1 (16.7)	2 (33.3)	1 (16.7)	3 (50)	6 (100)	6 (100)	5 (83.3)	2 (33.3)	3 (50)
*Enterobacter aerogenes*	3	–	–	–	–	–	–	1 (33.3)	3 (100)	3 (100)	2 (66.7)	1 (33.3)	0
Citrobacter spp.	4	4 (100)	3 (75)	2 (50)	2 (50)	2 (50)	2 (50)	4 (100)	4 (100)	4 (100)	3 (75)	3 (75)	3 (75)

*Amc* amoxicillin+ clavulanic acid, *Cip* ciprofloxacin, *Gm* gentamicin, *Sxt* sulfamethoxazole + trimethoprim, *Trm* trimethoprim, *Te* tetracycline, *Amp* ampicillin, *Cf* cephalothin, *Fox* cefoxitin, *Cro* ceftriaxone, *Su* sulfisoxazole, *ND* not done

Among 12 *Salmonella* isolates investigated in this study, 7(58.3%) were resistant to tetracycline and cefoxitin. Resistance in *Salmonella* isolates to more number of antimicrobials was observed in isolates obtained from rivers than those from hospital WWSs. *Klebsiella pneumoniae* strains isolated in the current study were resistant to several antimicrobials compared to *K. oxytoca* strains. Majority of *E. cloaceae* strains isolated in the current study were resistant to most of the betalactam antimicrobials like cefoxitin (second generation cephalosporin) in addition to ampicillin and cephalothin except a single isolate from Little Akaki river. Interestingly, two *E. cloaceae* isolates obtained from TASH WWS were resistant to most of antimicrobials tested including ciprofloxacin and ceftriaxone (third generation cephalosporin). On the other hand, the 3 *E. aerogenes* isolates were susceptible to majority of the antimicrobials examined. Similarly high rate of resistance to antimicrobials like amoxicillin + clavulanic acid, tetracycline, ciprofloxacin and ceftriaxone was recorded for the 3 *Citrobcter* strains isolated in this study (Table 3).

### Antimicrobial resistance pattern of species of Enterobacteiaceae isolated from wastewater samples and downstream water bodies

In general, diverse phenotypic resistance pattern was observed for majority of the species of Enterobacteriaceae isolated in this study. Overall, the highest rate of MDR was detected in two *Citrobacter* isolates obtained from TASH WWSs in which both isolates were fully resistant to all 12 antimicrobials tested. A single isolate obtained from Addis Ababa Abattoir was also resistant to 9 antimicrobials tested. Similarly, 2 *Enterobacter colaceae* isolates from TASH WWSs also exhibited high level of MDR including resistance to ciprofloxacin and third generation cephalosporin. Two *E. coli* isolates from TASH WWS were also MDR to 11 of 12 antimicrobials tested. In general, multi-drug resistance is more commonly observed in *Citrobacter, Enterobacter* and *E. coli* isolates and it is more common in isolates obtained from hospital WWSs particularly TASH compared to other sources. Majority of *Salmonella* isolates in the current study were resistant to less number of antimicrobialscompared to other species. Three isolates from the hospital and Addis Ababa abattoir WWSs were resistant to only two antimicrobials whereas majority of isolates from downstream water bodies were resistant to more number of antimicrobials (Table 4).

**Table 4:** Antimicrobial resistance patterns of bacteria isolated from various wastewater samples and tested for susceptibility to 12 antimicrobials

Bacterial species (Total No. tested)	Place of collection	Resistance pattern	No with this resistance-pattern	Total No. of antimicrobials to which isolate is resistant
Citrobacter spp. *(4)*	TASH	Amc,Cpr,Gm,S, Sxt, G, Tmp, Te, Amp,Cf, Fox, Cro,Ctx	2	12
	Kality WWTP	Amc,Cpr,G,Te,Amp,Cf,Fox,Cro,	1	9
	Little Akakiriver(1)	Amc, Te, Amp, Cf, Cro	1	5
*Enterobacter aerogenes (3)*	Little Akaki river	Amc,Te,Amp,Cf,Fox,Cro, Ctx	1	7
	AA abattoir	Amc,S,Amp, Cf	1	4
	AA abattoir	Amc,Amp,Cf, Fox	1	4
*Enterobacter cloacae*(6)	TASH	Amc,Cpr,Sxt,G,Tmp,Te,Amp,Cf, Fox,Cro	2	10
	Menilik II hospital	Amc,Amp,Cf	1	3
	Kebena river	Amc,Amp,Cf, Fox	1	4
	Kality WWTP	Amc,Amp,Cf, Fox	1	4
	Little Akaki river	TE,Amp,Cf	1	3
*Escherichia coli(18)*	TASH	Amc, Cpr, S,Sxt, G, Tmp, TE, Amp, Cf, Fox,Cro	2	11
	TASH	Amc,Tmp, Te, Amp, Cf	1	5
	TASH	Amc,Cpr,Amp, Cf	1	4
	Menilik II hospital	Amc, Cpr,S, Te, Amp, Cf, Fox, Cro	1	8
	Menilik II hospital	Amc,Cpr,S,Sxt,G,Tmp,Te,Amp	1	8
	Menilik II hospital	Amc,Cpr, Amp, Cf	1	4
	AA abattoir WWS	Amc,Amp, Cf, Fox	1	4
	AA abattoir WWS	S,Sxt,G,Tmp, Te,AMP, Cf	1	7
	AA abattoir WWS	Amc,S,Sxt,G,Tmp, Te,Amp,Cf, Fox	1	9
	AA abattoir WWS	Amc, Te, Amp, Cf, Fox	1	5
	Kality WWTP	Amc,Amp,Cf, Fox	1	4
	Kality WWTP	Te,Amp, Cf	1	3
	Kebena river	Te,Amp, Cf	1	3
	Little Akaki river	Amc,S,Sxt,G,Tmp, TE,Amp,Cf, Fox	1	9
	Little Akaki river	Amc,TE,Amp,Cf, Cro	2	5
*Klebsiella oxytoca*(3)	TASH	Amc,Amp,Cf	1	3
	Menilik II hospital	Amc,Amp, Cf	1	3
	Little Akaki river	Amc,Amp, Cf	1	3
*Klebsiella pneumonia*(8)	Menilik II hospital	Amc,Amp,Cf,S, Sxt,G,Tmp,Te	1	8
	Menilik II hospital	Amc,Amp,S, Cf	1	4
	AA abattoir	Amc,Amp, Cf	2	3
	Kality WWTP	Amc,Cpr,Tmp, Amp,Cf, Fox	1	6
	Kebena river	Amc,Amp,Cf, Fox	1	4
	Kebena river	Te,Amp, Cf	1	3
	Little Akaki river	Te,Amp, Cf	1	3
Salmonella spp.(12)	TASH	Amp, Cf	1	2
	Menilik II hospital	Amp, Cf	1	2
	AA abattoir	Amp, Cf	1	2
	AA abattoir	Amp,Cf, Fox	1	3
	Kality WWTP	Amc,Amp,Cf, Fox	1	4
	Kality WWTP	Amp,Cf, Fox	1	3
	Kebena river	Amc,Sxt,Tmp, Te,Amp,Cf,Fox	1	7
	Kebena river	Te,Amp,Cf	1	3
	Kebena river	Amc,Te,Amp,Cf, Fox	1	5
	Little Akaki river	G,Te, Amp,Cf, Fox	1	4
	Little Akaki river	S,G,Te,Amp,Cf,Fox	1	6
	Little Akaki river	Te,Amp, Cf	1	3

*Amc* amoxicillin+ clavulanic acid, *Cip* ciprofloxacin, *Gm* gentamicin, *Sxt* sulfamethoxazole + trimethoprim, *Trm* trimethoprim, *Te* tetracycline, *Amp* ampicillin, *Cf* cephalothin, *Fox* cefoxitin, *Cro* ceftriaxone, *Su* sulfisoxazole

### Extended spectrum betalactamase production by resistant isolates

Of the 22 isolates of enterobacteriaceae tested using double disc synergy test, only six (27.3%) were shown to produce ESBL. These isolates were *K. pneumonia* and *E. coli* isolated from WWS of Menilik II Referral hospital; Citerobacter species, *Enterobacter cloaceae*, *E. coli* isolated from WWS from TASH and *E. coli* isolate obtained from Little Akaki river. Proportion of isolates producing ESBL among the Enterobacteriaceae isolates examined and relative distribution among different species is shown in Table 5.

**Table 5:** Rate of occurrence of extended spectrum betalactamase production among Enterobacteriaceae using double disc synergy test

Bacterial species	DDST result		% positive	Collection site for positive isoltes
	No. tested	No. positive		
Citrobacter spp.	3	1	33.3	TASH
*E. coli*	11	3	27.3	2 from Menilik II Referral Hospital and TASH, 1 from Little Akaki river
*Enterbacter cloaceae*	4	1	25	TASH
*Klebsiella pneumonia*	3	1	33.3	Menilik II Referral Hospital

### Carbapenemase production

Modified carbapenem inactivation assay was conducted for 28 different Enterobacteriaceae which exhibited resistance to ampicillin, cephalothin, cefoxitin and ceftriaxone to examine if the isolates also produce carbapenemase enzyme. None of these isolates were shown to produce carbapenemase.

## Discussion

High rate of detection of *E. coli* compared to other species is not surprising as it is one of the commensal organisms commonly available in gastrointestinal tract of humans and animals. Contamination of wastewater from hospital and abattoir can easily pollute the receiving water bodies with potential multi-drug resistant pathogenic *E. coli* strains. Although this study did not investigate if the isolated *E. coli* strains in the current study were pathogenic or not, high rate of MDR was detected in majority of the isolates. Similarly *E. coli* was detected from the effluents of hospital wastewater in South Ethiopia [22]*.*

Resistance of *E. coli* to various antimicrobials, particularly to major betalactam antimicrobials and ciprofloxacin was high in isolates obtained from WWSs from hospital and Addis Ababa Abattoir compared to those obtained from downstream rivers and Kality WWTP. The finding of high rate of resistance among *E. coli* from hospital and abattoir WWS, is undoubtedly due to high selection pressure for resistant strains in the hospital and abattoir facilities because of excessive use of antimicrobials particularly in hospitals. Similar high rate of resistance among *E. coli* strains to betalactams from clinical, environmental origin and food from hospital has been reported in previous studies in different countries [23–26]. Since major mechanism of resistance to betalactam antimicrobials is due to production of betalactamase enzymes encoded by genes carried on various plasmids, these *E. coli* isolates can contribute to horizontal transmission of resistance genes to other bacterial species in the wastewater and receiving downstream water bodies [27].

Salmonella spp. were isolated from all sampling sites; however, detection rate was higher in samples from rivers compared to the hospital and abattoir WWSs. This is probably due to contamination of rivers by fecal materials of food animals or humans in addition to contamination with hospital and abattoir wastewater. *Salmonella* usually occurs in gastrointestinal tract or feces of various animals like cattle, poultry, small ruminants and dogs [28–30] which can easily access the rivers through direct discharge from the farms or through flood during rainy season. Serotyping of *Salmonella* isolates revealed serovars that commonly occur in food animals and cause human salmonellosis in Ethiopia and elsewhere like *S*. Newport and *S*. Typhimurium [30–32]. To our knowledge, *S*. Adelide is reported for the first time in Ethiopia in this study. *Salmonella* Adelide has recently been reported to cause multistate outbreak of human salmonellosis in USA [33].

High rate of resistance to ampicillin, cephalothin and tetracycline in *Salmonella* isolates is in line with findings for *Salmonella* isolates from food animals and humans in Addis Ababa [29, 31]. Vegetables sold in Addis Ababa market were shown to be contaminated by *Salmonella* in previous study [34]. Resistance to tetracycline is particularly high presumably due to widespread use of oxytetracycline in livestock production in the country [35]. Contrary to previous report of MDR strains of non-typhoidal *Salmonella* causing invasive disease in young children from TASH [36], a single *Salmonella* isolate resistant only to ampicillin and cephalothin was detected in the current study in WWS from TASH. However, this may not represent the real situation of the hospital environment as sampling was done only twice in this study. *Salmonella* isolates from rivers were resistant to more number of antimicrobials compared to those from hospitals and abattoir in the current study. This is probably due to contamination of rivers with other sources or due to acquisition of resistance genes from other bacteria in the river. Previous study in Addis Ababa showed that some dairy farms in Addis Ababa are contaminated with multi-drug resistant *Salmonella* and the drainage from these farms may access the rivers [29, 37].

Overall occurrence of resistance among *Klebsiella* isolates obtained from abattoir was high compared to isolates from other sources particularly to betalactam antimicrobials and sulfisoxazole. *Klebsiella pneumonia* from hospital environment is widely known to carry genes coding for resistance to several antimicrobials including those producing ESBL and *Klebsiella pneumonia* carbapenemase (KPC) [38, 39].

Detection of MDR strains of Citrobacter species from TASH wastewater is in line with high rate of antimicrobial use in the hospital and previous reports also showed MDR Eneterobacteriaceae in TASH causing severe morbidity and mortality in hospitalized patients [40].

The two *E. cloaceae* isolates from TASH were both MDR to several antimicrobials. Both *E. aerogenes* and *E. cloaceae* are causes of opportunistic nosocomial infections responsible for outbreaks [41]. *Enterobacter* is also claimed to serve as the reservoir of antimicrobial resistance genes. They are known to acquire numerous mobile genetic elements which contribute to fitness of the organism to colonize several environments and hosts. Horizontal transfer of resistance genes from *Klebsiella pneumoniae* is implicated to be the main reason for wide occurrence of MDR *Enterobacter* species in hospital environment [42]. Therefore, detection of such MDR strains in hospital WWSs is an indication of risks associated with potential life-threatening infection as well as possibility of downstream dissemination of MDR isolates and MDR conferring genetic markers to other bacterial communities. A few *Enterobacter* strains isolated from the rivers and Kality WWTP were not resistant to several antimicrobials unlike those isolates from hospital wastewater. This is presumably due to dilution effect of the environmental sources of *Enterobacter* species which are not exposed to antimicrobials since the current study protocol was not designed to pick MDR isolates selectively. *Enterobacter cloaceae* is ubiquitous in terrestrial and aquatic environments [43].

In general, high level of MDR as high as resistance to all 12 tested antimicrobials in Citrobacter species from TASH wastewater, and 11 antimicrobials in *E. coli* isolated from wastewater sample from the same hospital were recorded in this study. Similarly, resistance to 8 antimicrobials in *E. coli* from Menilik II Referral Hospital wastewater and 9 antimicrobials in *E. coli* from Addis Ababa Abattoir were recorded. This shows that wastewater from these sites particularly TASH is serving as a major hot spot for MDR isolates and resistance genes. Interestingly a single Citrobacter species isolated from Kality WWTP was also resistant to 9 antimicrobials including ciprofloxacin and ceftriaxone. This isolate probably originated from TASH or other health facility in the way towards treatment plant. *E. coli* and *Citrobacter* are known to be the major causes of nosocomial infection in TASH [40].

Dissemination of such MDR strains carrying resistance genetic markers may impose high risk of spread of resistance genes. The level of MDR in isolates obtained from Kality WWTP and Little Akaki River was not as high as the one obtained from hospitals. However, low level of MDR in strains isolated in this study may not gurantee absence of MDR bacteria in downstream water bodies, since the isolation procedure employed was not exhaustive as only single colony was picked from a plate.

The reason why majority of ESBL producers were derived from hospital wastewater could be due to the fact that selection for ESBL producing organisms occurs easily in hospital environment due to overuse of multiple antimicrobials in patients admitted to hospital and suffering from various nosocomial infections. The observed MDR phenotype to betalactams including third generation cephlosporins in the isolates which are negative for ESBL production could be due to production of broad spectrum betalactamases, inhibitor-resistant betalactamases and cephalosporinase over production [19].

In this study, carbapenemase production was not demonstrated in any of the isolates which is contrary to the report from other country where crbapenemase producing entrobacteriaceae was reported in hospital wastewater [44]. The low usage rate of carbapenem antimicrobials in hospitals in Ethiopia may be the reason for not detecting carbpenemase production in the current isolates.. Recent report on Enterobacteriaceae isolated from children suspected of septicemia and urinary tract infections in Addis Ababa showed carbapenem resistant enterobacteriaceae (CRE) in 12.1% of the isolates [45]. As the nuber of samples investigated in this study is small, absence of carbapenemase production in isolates in the current study doesn’t rule out presence of CRE strains in WWSs in the current study area at least for those from hospitals.

## Conclusions

This study demonstrated antimicrobial resistance among Enterobacteriaceae in wastewater from hospitals and Addis Ababa Abattoir as well as receiving downstream water bodies. High level of resistance was particularly detected in *Citrobacter* and *E. coli* isolates from hospital wastewater. The use of downstream rivers contaminated with MDR bacterial pathogens poses public health risk to those using these rivers for irrigation and residents of Addis Ababa who use the vegetables grown by these farmers. These MDR bacterial species may also transmit their resistance genes to other pathogenic or commensal organisms in the water bodies and also in the gut of humans and animals further increasing spread of antimicrobial resistance. Therefore, hospitals and abattoir wastewater should be treated before being released into the environment. Further studies should be conducted in the region to reveal healthcare liquid waste management and microbiological quality of effluents discharged into receiving environment.

## Data Availability

The authors confirm that all the data are included in the manuscript.

## Abbreviations

BPW: Buffered peptone water
CRE: Carbapenem resistant Entrobacteriaceae
DDST: Double-disc synergy test
ESBL: Extended spectrum betalactamase
MDR: Multi-drug resistance
RVB: Rappaport-Vassiliadis broth
TASH: Tikur Anbessa Specialized Hospital
TTB: Tetrathionate broth
WWS: Wastewater sample
WWTP: Wastewater treatment plant
XLT4: Xylose Lysine Tergitol 4
